# “Covid Just Amplified the Cracks of the System”: Working as a Frontline Health Worker during the COVID-19 Pandemic

**DOI:** 10.3390/ijerph181910178

**Published:** 2021-09-28

**Authors:** Karen Willis, Paulina Ezer, Sophie Lewis, Marie Bismark, Natasha Smallwood

**Affiliations:** 1 School of Allied Health, La Trobe University, Bundoora, Melbourne, VIC 3083, Australia; P.Ezer@latrobe.edu.au; 2 College of Health and Biomedicine, Victoria University, Footscray, Melbourne, VIC 3011, Australia; 3 Faculty of Medicine and Health, University of Sydney, Camperdown, NSW 2006, Australia; Sophie.Lewis@sydney.edu.au; 4 Centre for Health Policy, Melbourne School of Population and Global Health, University of Melbourne, Parkville, VIC 3050, Australia; mbismark@unimelb.edu.au; 5 Department of Respiratory Medicine, The Alfred Hospital, 55 Commercial Road, Prahran, VIC 3004, Australia; Natasha.smallwood@monash.edu; 6 Department of Allergy, Immunology and Respiratory Medicine, Central Clinical School, The Alfred Hospital, Monash University, Melbourne, VIC 3004, Australia

**Keywords:** Australia, COVID-19, extreme work, frontline health workers, health professionals, qualitative

## Abstract

The COVID-19 pandemic has intensified existing workplace stresses and created new challenges for people working on the healthcare frontline, including rapid workplace changes and increasing uncertainty at work, along with fear of contracting the virus. The purpose of this study is to examine the workplace challenges described by Australian frontline health workers during the pandemic. Drawing on a national online anonymous survey of 9518 healthcare workers, we analysed free-text responses to the question: “What did you find to be the main challenges that you faced during the pandemic?” A qualitative content analysis was undertaken. We identified five key themes relating to participant experiences of workplace challenges. These were: Work-life imbalance; Following orders or caring for patients; Unpredictability, disruption, and inconsistency at work; The right to be safe at work; and (Un)preparedness in the workplace. We argue that working during the COVID-19 pandemic illuminated existing occupational health and safety issues that have serious implications for job satisfaction, health workforce retention, and ultimately, patient care.

## 1. Introduction

Frontline healthcare often occurs in a high-pressure work environment where health workers make multiple decisions and perform numerous actions over short timeframes and sometimes with limited resources. Crisis events, such as the COVID-19 pandemic, can exacerbate pre-existing workplace stresses and create new challenges for frontline health workers, including increased workloads, unfamiliar tasks outside their usual scope of work practice [1], disruption of established teams, and rapidly changing policies and procedures. Together, these changes pose significant threats to workplace health and safety [2] with potentially adverse consequences for both patients and health workers.

Globally, Australia has been commended for its swift and effective response to the pandemic with just over one thousand deaths in a population of 25 million people between 27 May 2020 and 1 September 2021 [3]. By limiting the movement of people into and within Australia and implementing stringent lockdowns when outbreaks occur, the spread of the virus has been less than in many countries. Despite experiencing many fewer health worker infections and deaths than many other countries, health workers in Australia are disproportionately infected [4] and have experienced significant psychological, social, and occupational harms during the pandemic [5,6,7].

In this paper, we examine the workplace challenges described by frontline health workers during the pandemic in Australia using free-text responses from a large national survey. These challenges are important to understand and address, given the vital role that health workers play during pandemics and other crises [8] and the potential consequences for patient care if health workers become overwhelmed or disengaged, or leave the profession [9].

### The Challenges of Healthcare Workplaces

Healthcare workplaces in many countries have focused on improving efficiency and performance, under the influence of new public management strategies. However, as Scambler [10] notes, the consequent effects on preparedness for crises, such as a pandemic, have been severe. The emphasis on efficiency, privatisation, accountability, and austerity all contribute to a work environment where there is little “organisational slack” [11] and where work becomes “intensified”, with potentially negative consequences for job satisfaction among health workers and for patient care [12]. The experience of such intensified work conditions can be described as “extreme work”. While often encapsulated as excessive work hours (usually defined as more than sixty hours per week), extreme work can also occur where the workflow is unpredictable, work duties are expanded, and workplace psychological or physical risk increases [13,14,15]. As many health professionals would attest, extreme work can be simultaneously challenging and rewarding. It can also lead to fatigue and burnout, with negative ramifications for individual frontline health workers and patient care [16,17].

A further complexity of contemporary frontline work, exacerbated during crises such as pandemics, is the increase in the permeability of boundaries between work and non-work and the challenges for frontline health workers in navigating work and non-work activities [18]. Boundary management practices are usually based on organisations’ or individuals’ preferences for segmentation (separate work and non-work domains) or integration (overlapping work and non-work domains) [19,20]. However, workplace boundaries have become more tenuous as contemporary workplaces and work practices have become increasingly flexible [21], particularly during the pandemic when technologies such as telehealth were rapidly adopted, enabling healthcare to be delivered remotely. This flexibility blurs the demarcation between work and non-work spaces or time but may also illustrate fundamental inequalities in choice and control over one’s work [22].

A feature of working at the frontline of healthcare that posed particular dilemmas during COVID-19 is that challenging and often moral decisions must be made within an organisational context of extreme work. Although frontline health workers make difficult decisions throughout every working shift, senior management is often removed from the everyday social, moral, and ethical issues faced by health workers on the frontline [23], leading to workers feeling trapped between organisational and patient needs [24]. One of the struggles that has been amplified during the COVID-19 pandemic is the extent to which patient care might be compromised due to rapidly changing practices, the increasing complexity of health professional responsibilities, and/or lack of resources. The extent to which frontline health workers feel compromised in the decisions and actions they are forced to take during crises is central to their wellbeing at work [23,25]. Though there has been extensive public messaging about how difficult frontline healthcare work has been during the pandemic, there has been very little research exploring the experiences of those working on the frontline.

## 2. Materials and Methods

### 2.1. Methodological Approach

Our research question was “what were the workplace challenges experienced by frontline healthcare workers during the COVID-19 pandemic?” We were keen to identify what the challenges for health workers were during a time of crisis, and whether tensions such as those identified above were evident during the pandemic. Our team comprised frontline healthcare clinicians and social science academics. We took a qualitative descriptive approach, well suited to examining the perspectives and worldviews of those most involved in a phenomenon [26] and when the research topic requires a rich description of an experience of an event [27].

### 2.2. Participants and Design

Our data were collected as part of the Australian COVID-19 Frontline Health Workers Study, an anonymous, online, national survey conducted between 27 August and 23 October 2020 that investigated the psychosocial impacts of COVID-19 on health workers. Details of this survey method, including sampling and recruitment, have been reported, along with some of the findings from quantitative data [28]. Survey participants included all those who identified as “frontline” health workers, such as medical, nursing, allied health, medical laboratory, administrative, and other support staff.

The survey included four free text questions in addition to quantitative questions and psychometric measures, enabling participants to provide information about varying aspects of their experience. These questions were:

1. What do you think would help you most in dealing with stress, anxieties, and other mental health issues (including burnout) related to the COVID-19 pandemic?
2. What did you find to be the main challenges that you faced during the COVID-19 pandemic?
3. What strategies might be helpful to assist frontline healthcare workers during future crisis events like pandemics, disasters, etc.?
4. Is there anything else you would like to tell us about the impact of the COVID-19 pandemic or regarding supports that you feel are useful for well-being?

Each of these questions generated differing information, and in this paper we focus on Question 2 to examine the workplace challenges as described by participants. There were 6679 responses to this free text question (from a total of 9418 survey responses overall). With no pre-determined length of response, nor a requirement to answer, participants could write as much or as little as they wished. Some wrote one line; many wrote 2–3 paragraphs.

### 2.3. Ethics

The study received ethics approval from the Melbourne Health Human Research Ethics Committee (HREC/67074/MH-2020 approved 20 August 2020). All participants provided consent online. Information for participants was provided on the survey website, indicating that data would be stored securely, that no identifiable data were being collected, and that care would be taken to ensure that data remained anonymous in reporting. As the survey was about the psychosocial impact of COVID-19, links to mental health resources were also provided on the survey website.

### 2.4. Data Analysis

Following Morgan [29], we undertook a qualitative content analysis of the data, focusing on “manifest content” [30] in line with the approach of qualitative description, where “staying close” to the data is emphasised [27]. Based on an initial reading of the first 100 responses, a code book was developed by 3 authors. The codebook categorised responses into 3 broad areas: challenges related to personal experiences, challenges related to work experiences, and challenges related to broader societal or political factors. Each response was coded, with up to three codes being applied per free text response. A brief description of each code was included in the codebook, and an iterative process was undertaken where additions were made to the codebook, as new ideas emerged from the data.

Once all responses were coded, the data within each code were sorted and compared within and across the codes identifying similarities and differences in ideas expressed to develop themes (see Table 1 below). Codes with similar or related ideas and concepts were grouped together into key themes [31,32]. For example, the theme about the right to be safe at work (see findings below) was drawn from the ideas presented in four main codes: Risk (defined as risk or fear of being infected at work), Safety (work safety not prioritised or valued), PPE (PPE availability, PPE rationing), and Stress and Anxiety (feelings of being unsafe). 

### 2.5. Rigour and Trustworthiness

Consistent with guidance from Elo et al. [33], data were organised systematically, with more than one investigator involved in coding and analysis. Three investigators separately coded free text responses systematically using Microsoft Excel, then met weekly to discuss and refine the codebook, which contributed to ensuring inter-rater reliability and trustworthiness of findings. In these weekly meetings, any differences in the interpretation of codes were discussed until a consensus was reached. At varying stages in manuscript preparation, all authors provided feedback on the “fit” between the codes, the findings, and the quotations used to illustrate findings.

## 3. Findings

### 3.1. Study Participants

A total of 6679 participants provided a free text response describing the challenges they faced. As shown in Table 1, they were drawn from across the range of health occupations and represented an even spread of age ranges, and while more women than men participated, this is broadly reflective of the demographics of the Australian health workforce [34]. 

### 3.2. Overview of Findings

Two thirds of the responses were coded as being about the challenges associated with work-related experiences, and it is these responses about the challenges at work that are the focus of this article. Our key finding is that the COVID-19 pandemic amplified existing workplace issues, widening “cracks in the system” that existed prior to the pandemic. As shown in Table 2, 5 key themes were identified in relation to the challenges described by participants. In some cases, codes overlapped (depending on the content of the coded response), and as indicated above, it was through an examination of the key ideas underpinning each code that the themes were developed.

In presenting these findings below, we include illustrative quotes with demographic information about the participants, including their occupation, workplace setting (where possible), gender, and age group. The inclusion of these characteristics conveys how widespread the challenges experienced were, across the health workforce. Participant quotes are italicised.

### 3.3. Increased Workload and Work-Life Imbalance

The onset of the pandemic brought increased difficulties in balancing work and personal lives, as workload increased and intensified. Healthcare workers described their sense of imbalance as COVID-19 permeated all facets of life:

*Having to think about COVID-19 24/7. All day every day at work it is at the front of my mind, seeing everyone in masks all day, seeing all the new things put in place (i.e., temp check, entries closed) and then to go home and talk about it with my family, and see it on the news. I find it challenging to not be able to stop thinking about it, like other people who aren’t in healthcare would be able to. It’s exhausting, especially because we have been dealing with it for over 6 months now.*

(Administrative Staff, Primary Care, female, age 20–30)

This pervasiveness was exacerbated as healthcare workers’ workloads increased, and workload became a challenge:

*The added workload of policy development and interpretation to my existing workload.*

(Nurse, Maternity, female, age 31–40)

*I have had to work incredibly flexibly, picking up workloads that would never normally be my role.*

(Nurse, Education, female, age 31–40)

In describing the intensification of work, participants wrote about a health system that was already under stress with high workloads and inadequate resourcing. In this context, responding to the increased demands at work due to the pandemic was extremely challenging:

*Workload went up, not down, and pre-COVID-19 it was already excessive.*

(Senior Doctor, Palliative Care, female, age 41–50)

*We have exactly the same resources as we did pre-COVID. Not having breaks and having to work 7–10 days straight and also the increased frequency of working shifts and night shifts. The system was already underfunded. COVID-19 just amplified the cracks of the system. I often came home feeling defeated and too tired to take care of my own physical and mental health.*

(Allied Health, Emergency Department, female, age 20–30)

Changes to the work setting were also described as challenges. Many of those working from home detailed difficulties with delivering patient care, and described difficulties managing their workspace at home, particularly if they had other family members at home. Some both had to undertake patient consultations and care for their family in their home while other kinds of support, such as childcare or family members helping with care, were unavailable. This led to participants feeling they were “*doing a bad job at home and at work*” (Senior Doctor, General Medicine, female, age 31–40), and this feeling is exemplified in the words of the following General Practitioner as she described the challenges she faced:

*Balancing part time work and the increase in administrative tasks, with ongoing parenting role and increased general household responsibilities which have risen above and beyond what I’ve previously had capacity for. […] The constant juggle of being not available to work whilst awaiting swab results, or symptom resolution from the “usual” viruses our children are exposed to (and it simply being not feasible to conduct Telehealth consultations at home with young children in the home).*

(General Practitioner, female, age 41–50)

There were differing views on the impact and benefits of working from home. Some participants described being encouraged to work from home while others were not, and some were forced to work from home when they would have preferred not to. The privileging of options for some workers but not others was suggestive of divisions emerging between co-workers. This was particularly mentioned by junior medical staff who were required to take on additional responsibilities despite their already heavy workloads and limited experience. The struggle they experienced as they felt left behind in the clinical setting to cover for more senior colleagues is exemplified in the following:

*Stress with having to bear increased load at work due to others who are working from home continuously/for weeks consecutively. As junior medical staff, we are expected to be at work so that the more senior medical staff can stay home, that can increase the amount of work during the day in hospital.*

(Junior Doctor, female, age 31–40)

The contention around working from home, and the assumption that such decisions were about whether employees could be “trusted” to work from home, is described by this Allied Health frontline worker:

*Not able to work from home for 100% of time, even though we are not seeing clients face to face, unless urgent. No reasonable explanation given as to why. If we can work for 50% of the time, we should be able to work for more hours at home if we have no face to face contact with clients. Maybe they do not trust us? It seems ludicrous that we have to wear a face mask while sharing an office and a face shield when we move along the corridors, when we could be working at home, where our risk of being infected is zero.*

(Allied Health, Community Health, female, age 51–64)

### 3.4. Following Orders or Caring for Patients

Healthcare workers described incongruence between what health managers expected of them in performing their daily roles and what they considered best practices for patient care. This incongruence has been reported in studies of other occupations where the “middle” strata are torn between labour and management [22], and in emerging findings on moral distress experienced by healthcare workers [25]. Alongside an expectation of rapid adjustment to new processes was the possibility that patient care or their own safety would be compromised. This inability to make decisions about patient care or personal safety became a threat to their identity as a health professional. As described below, this represented a major challenge:

*Having to implement (and promote) practices that I don’t believe in or are not evidence-based, or even ‘common sense’ based at times.*

(Nurse, Maternity, female, age 31–40)

Patient care was also raised as an issue by senior staff, and in the response below, a senior medical staff member describes this challenge and associated work conditions:

*Switching to remote consulting has been challenging and often leads to suboptimal care. … Drawing up protocols of how we would wind back and potentially stop chemotherapy treatments on our patients if the pandemic was to become overwhelming.*

(Senior Doctor, male, age 51–64)

Many health workers were critical of leadership within their organisation, and one of the contributing factors that challenged them was not being listened to when they expressed concern to their health managers about patient care:

*Managers not listening to changes required for patient care … at the front line.*

(Nurse, Emergency Department, female, age 41–50)

*Health workers’ concerns were ignored early on, especially relating to PPE supplies.*

(General Practitioner, female, age 31–40)

Participants thus described the “reality gap” between clinicians who were delivering patient care and their managers who were removed from the direct reality of doing so, resulting in a major challenge for many as they struggled with:

*An attitude which implied nurses just needed to work together better, deal with it, accept it and so on when expressing concerns or issues with the workload and demand on bedside ICU nurses by management/people who have not stepped foot in a COVID-19 positive room, worn the PPE for longer than a quick trial and truly experienced the reality of caring for a COVID-19 patient.*

(Nurse, Intensive Care Unit, female, age 20–30)

These experiences led many participants to feel that managers did not value them, particularly with regard to the expertise that they brought to their role, as indicated in the following two quotes by nurses:

*The healthcare workforce felt a bit like being in the army, and I’m not up for that. I feel like my strengths weren’t put to use, I didn’t have a voice, and was expected to ‘step up and step in line’. […] looking back and reflecting I just feel like I’ve been expected to work like a robot that can perform and get on board with anything... rather than a human with strengths and preferences. I’ve got a lot to offer if I’m allowed to work in ways that also work for me, rather than the sort of ‘one size fits all’ approach of hospital generally.*

(Nurse, gender not specified, age 31–40)

*Middle management lack of support and early pandemic direct criticism of concerns and questions and then as our questions and concerns became accepted, no validation, no apologies, no appreciation for attempting to be safe and as early identifiers of issues and inadequacies in our facility. Just personal avoidance. Being told that reminding social distancing and wearing masks made patients panic was so untrue, now to never see managers, or even know if and when they are on site is demoralising and makes ‘on-the-floor’ workers feel unvalued.*

(Nurse, female, age 51–64)

Pressures at work also came from patients or their family members who expressed their frustrations with visitor restrictions or new patient care processes to frontline health workers. The unrecognised impact of bearing the brunt of patient or family discontent was also described as a “reality gap” between frontline staff and their managers.

*Executive/management not understanding how stressful/how much abuse staff receive from patients when communicating these updates/changes to patients/family. They are very out of touch. Also poor communication to patients from the organization—leaving it up to individuals to deal with hostility and disappointment from patients themselves, with little/no support.*

(Administrative Staff, Surgical, female, age 20–30)

### 3.5. Unpredictability, Disruption, and Inconsistency at Work

Participants also wrote about being challenged by increased unpredictability, disruption, and inconsistent or confusing messaging particularly pertaining to guidelines and rapid change at work:

*It feels like every shift is unpredictable.*

(Junior Doctor, Emergency Department, female, age 31–40)

The rapidity of changes to regular workplace processes and clinical practices was also disruptive:

*We all of a sudden had to flip and rethink how we did everything, like the direct front line areas but not with the same level of support.*

(Nurse, Surgical, female, age 51–64)

Unfamiliar processes, a lack of available COVID-19 training, and redeployment to other areas of healthcare caused some to feel unprepared in their work with challenges being described as:

*Being redeployed to other areas with little time for orientation to the COVID processes in place.*

(Nurse, Intensive Care Unit, female, age 51–64)

The ever changing and sometimes conflicting guidelines made health workers’ work more confusing:

*Negotiating all the different and dynamic work instructions and protocols was [very] hard as they change every day.*

(Senior Doctor, Anaesthetics, female, age 41–50)

*Keeping up to date with processes, criteria, all the programs that get rolled out. Rules re PPE, ward transfers, visitors, its overwhelming and changing regularly. Every single day, there is something new to take on board!*

(Nurse, Emergency Department, female, age 31–40)

Those in leadership positions also commented on changes to the work environment, which made it more difficult to give clear directives. The challenges they faced included:

*Keeping current in my managerial role to ensure everybody is up to date with latest information and work place plans.*

(Administrative Staff, Primary Care, female, age 51–64)

Words such as “frustrated” and “exhausted” recurred often, indicating the emotional dimensions of the challenges they were experiencing, as is indicated in these responses:

*As we’ve discovered more about COVID-19, the guidelines and advice have constantly changed and it has been my job to stay abreast of all of this which in itself is challenging, but it’s also my job to educate other health workers about these changes. I am often on the brunt of exhausted, frustrated and even angry responses to yet another update or change in practice.*

(Nurse, Education, female, age 31–40)

*Being in charge of shifts in the ICU during both waves, there were so many mixed messages and muddled policies and processes from medical and nursing directors and executives that bedside nurses were asking me what to do and I really couldn’t give a clear directive. It was immensely frustrating.*

(Nurse, Intensive Care Unit, female, age 31–40)

Many participants described information overload as a result of the many changes to guidelines and work procedures. It was difficult to stay on top of messages, both due to the volume of information and the multiple sources of information:

*Inundated with information that was sometimes conflicting.*

(Paramedic, male, age 51–64)

*Over-communication and constant emails [from] the organisation meant that a lot of info doesn’t get read because who has the time.*

(Nurse, Surgical Female, age 20–30)

*Constant barrage of communication from multiple different workplaces using email and apps with rapidly changing guidelines that we were expected to be the experts on by other professions at work.*

(Junior Doctor, Anaesthetics, female, age 20–30)

Others, however, criticised the lack of information that was provided:

*At [name of hospital], they have failed to communicate, point blank refusing to provide communication, and on the few occasions that they did provide communication, providing misleading information … Or holding an hour’s briefing, saying nothing and then effecting major changes to the way we work within 2.5 working days of the briefing.*

(Dietician, Rehabilitation, female, age 41–50)

Both over-communication and lack of communication gave many a sense of poor leadership at the height of the pandemic response; and again the “reality gap” was evident in the challenges described:

*Very autocratic leadership from hospital executive, things imposed/taken away without consideration on morale, necessary infrastructure changes no implemented/poorly done, plan to cut ED staff because ‘numbers down’ without consideration of how much more difficult the job has become.*

(Senior Doctor, Emergency Department, female, age 41–50)

### 3.6. The Right to Be Safe at Work

Healthcare workers were extremely concerned about the level of risk in the workplace. Sometimes this was due to their workspace layout that did not allow for social distancing measures:

*We don’t have work spaces designed with social-distancing in mind.*

(Junior Doctor, Hospital Aged Care, female, age 20–30)

The factor that most exemplified the exposure to risk was the lack of access to appropriate PPE, and this was a recurring issue throughout descriptions of the challenges faced:

*The main challenge for me has been wanting PPE and infection control measures put in place much sooner, and feeling anxious until they were finally put in place.*

(Radiographer, Emergency Department, female, age 41–50)

The issues with adequate and timely access to PPE exemplified how participants felt they were valued by their organisations. The lengthy quotes below by two senior doctors encapsulate the resultant lack of confidence in the safety provisions for staff at work:

*Lack of appropriately fitted PPE—not enough N95 masks and no fit testing which should be mandated. I have no confidence that the N95 masks fit me properly. I don’t think my organisation was totally honest regarding PPE … We were told no surgical masks if looking after ‘low risk patients’ on coronary care and surgical mask only for looking after COVID patients who were not coughing and not having an aerosolising procedure. I gather these guidelines have now been changed due to the high numbers of health care worker infections—this was obvious from the beginning and has made it hard to trust hospital administration. There has been no attempt to get us proper fit testing—this means we’re working in an unsafe work place.*

(Senior Doctor, Coronary Care, female, age 51–64)

*The lack of care and support the hospital actually showed the doctors and nurses working on the COVID ward. The damage has been done. I was bullied and so were many of the staff on the COVID ward into wearing inappropriate PPE. They only changed practice because the hospital had an outbreak and it made it into the media. They showed little concern when the pandemic started for workers and cared little. […] We were seeing COVID positive patients in a surgical mask and a plastic apron initially when the evidence showed this was dangerous.*

(Senior Doctor, General Medicine, male, age 41–50)

Many felt that safety was not considered a priority by their management or the Australian government, and this is evident in the responses below:

*My main point of being upset was being told to enter the room post disconnection [from the ventilator] with a cloth gown and when I asked for the yellow waterproof gown I was told ‘we are saving them’. I felt I my safety and life was not worth wasting a gown on. I seriously felt like quitting.*

(Nurse, Intensive Care Unit, female, age 51–64)

*Trying to convince my community nursing bosses that providing a simple mask to our in-home patients to wear whilst we were attending them was a wise investment in nurse safety. This was refused. Waiting for masks to become mandatory so that we would be provided masks to use whilst doing in-home care in suburbs with high levels of COVID-19. [The Government] basically did not give a damn about how community nurses, occupational therapists, etc., were going to be protected.*

(Nurse, Primary Care, male, age 51–64)

*With all the news about fit testing, you wonder whether the PPE in place is actually sufficient and properly applied—particularly as in the early stages, we were told that surgical masks are sufficient, could wear same PPE on SCOVID/COVID ward between patients—clearly not the case, but if that’s the ‘expert advice’ that was guiding policy early, it’s hard to believe that we are being adequately protected.*

(Physiotherapist, Intensive Care Unit, female age 31–40)

### 3.7. (Un)preparedness in the Workplace

For many healthcare workers, the greatest challenge they felt was a lack of crisis (pandemic) preparedness in the workplace. Overlapping with themes above relating to safety at work, overwork, and the need for clear leadership, healthcare workers wrote about the lack of preparedness across the healthcare system. This was often framed in terms of management responsiveness versus preparedness:

*A sense that no one was a few steps ahead/leading.*

(Junior Doctor, Hospital Aged Care, female, age 20–30)

In addition, that:

*Everything is reactive instead of planning. We only do things after an event occurs. There should have been more planning.*

(Social Worker, General Medicine, female, age 20–30)

That lack of preparedness was linked to information flow, along with concerns about preparedness as the pandemic was unfolding. The concern about lack of information is exemplified in the following:

*The fact that we are in a pandemic does not seem to be at the forefront of thinking. At my team meetings, we do not discuss the COVID-19 pandemic weekly (numbers, use of PPE, what is happening in the hospital etc.). Information is provided on a need-to-know basis and at one level it feels as if it is business as usual.*

(Allied Health, Primary Care, female, age 51–64)

Communication and policy advice, and even whether organisations acted sufficiently quickly on evidence about transmission of the virus, all contributed to staff members feeling that their safety continued to be compromised due to a lack of preparedness at the healthcare system level, as discussed by a senior doctor:

*Poor planning, poor communication and poor advice at national, state and hospital levels. The hospital based outbreaks, transmission from asymptomatic patients, potential for aerosol spread in poorly ventilated environments etc. was all predictable at the start of the pandemic and yet the approach was to guess that only droplet spread was important, to use this guess as the basis for all policy rather than the more cautious approach of using higher levels of protection due to the significant possibility of aerosol spread.*

(Senior Doctor, male, age 41–50)

Exposure to the potential risks also seemed to highlight the lack of preparedness in settings which had been considered to be “non-COVID” spaces but where outbreaks still occurred, as described by this allied health frontline worker:

*My workplace had a COVID plan for hospital admissions, however the outbreak I was exposed to was unplanned. It became evident there had been a feeling of invincibility, as there was no clear plan in place for dealing with an outbreak on a ‘non-COVID’ ward.*

(Occupational Therapist, Hospital Aged Care, female, age 20–30)

A lack of resources or equipment such as PPE and ventilators was also discussed as being indicative of workplace preparedness:

*Rationing of treatment because of a lack of ventilators and ECMO [Extracorporeal Membrane Oxygenation equipment].*

(Senior Doctor, Respiratory Medicine, male, age 31–40)

Frontline health workers in primary care highlighted that the lack of preparation was systemic, and that this required a considerable investment of time, finances, and energy as they grappled with the challenges of the pandemic. The following response from a general medical practitioner working privately is indicative of the challenges faced:

*I’m a practice owner. There have been 2 waves of challenges coinciding with the 2 waves of infections. I wrote my first pandemic protocol for our clinic in January—at that stage I was doing so blind! Guidance came far too late—thankfully we acted independently and did so quickly. We adapted our clinic and so far have not had 1 single positive case step foot in our door. We have purchased our own PPE and set up every GP (we are a clinic of 16 doctors) to work from home. It was a massive challenge and there were weeks of barely any sleep as I wrestled with how to keep everyone (my staff, my patients, my family and myself safe).*

(General Practitioner, female, age 41–50)

Private practitioners also highlighted the increase in the presentation of mental health issues. Mental health services in Australia, as in many other countries, are known to be underfunded with considerable unmet needs [35]. COVID-19 exacerbated what some have called a mental health crisis, and those working in primary care called for health system reform:

*Dramatically increased incidence of mental health issues in the community—often in the primary care setting where we are already tight-for-time, there is just not enough time to do justice to the patient’s concerns and symptoms. It is only reasonable that patients turn to GPs for mental health support—this is not a rap for making more money but please review GP remuneration because otherwise more and more GP clinics will have to shut down due to becoming financially unviable ventures.*

(General Practitioner, female, age 31–40)

## 4. Discussion

Frontline health workers’ work environment can be described as “extreme work” [12,13], and their experiences of work during COVID-19 exacerbated work-life imbalance, impacted their personal lives, and presented a range of work-based challenges. In recounting the challenges they experienced during the COVID-19 pandemic, frontline health workers identified that their concerns reflected broader workforce concerns as work intensified and at the same time became more challenging. In amplifying the already existing “cracks in the system”, frontline health workers described struggling to straddle the divides they usually navigated. Their struggles can be seen in their descriptions of competing ideas and competing interests, as they navigated a rapidly changing work environment. Similar to other studies [6,36], our findings indicate that these negative experiences of work occurred across all groups of frontline health workers who experienced struggles between work versus home, between organisational interests versus patient interests, and between guidelines and policies versus what works on the ground.

Healthcare workers’ descriptions of the challenges they faced during the COVID-19 pandemic indicate not only how they were challenged at work, but also the extent to which the pandemic made visible the negative effects of existing workplace arrangements [10]. There are striking parallels between the experiences of these frontline workers and those previously reported in other “essential services” such as food services where risk is high and control over the work environment is low [22]. Particular tensions were evident in challenges about working from home and the provision of a safe working environment, exacerbated by inconsistencies in decision making. In exposing cracks in the health system, it is evident that a key challenge for frontline health workers is the experience of “extreme work” [12] in its many forms—excess work hours, unpredictable workflow, expanded work duties, and workplace risk—all of which were noted by participants. International research indicates similar findings: that health workers working throughout the pandemic faced increased workplace stressors such as longer shifts [37], increased workload [17,38], and increased work demands [39]. COVID-19 accelerated the rise of the virtual provision of health care, in many cases transforming health care delivery [40]; however, working remotely (i.e., telehealth) further impacted the work-life imbalance of healthcare professionals as they navigated work-home boundaries, along with inconsistency about what services could be provided remotely and ambiguity about whether health workers needed to be onsite at work, even if they were delivering telehealth.

The evidence from our study is reflective of other findings about the challenges faced by health workers [41,42]. For example, Liu, Chen, and Li argue that organisational responses are needed to address health workers’ concerns during crises events [43]; Ardebili et al. call for urgent attention to health workers’ mental health needs [41]; and Arnetz et al. [44] found that workplaces’ responses to the pandemic was a major contributor to stress experienced by nurses. Pervading many of the challenges experienced by participants in our study was what we have termed the “reality gap” between health management and frontline staff, drawing attention to the moral dimensions of the work of frontline health professionals and the tensions in adhering to changing workplace guidelines. When forced to follow new guidelines and procedures that seemed incompatible with best practices for patient care or their own wellbeing, they became caught between being a good worker, a good colleague, and a good professional to their patients. Feeling unheard and undervalued by managers, who were often removed from the frontline, amplified their frustrations. Throughout the data, there is evidence of suggestions for overcoming challenges experienced at work. They include the need for more supportive management, greater control over their work conditions, and clearer communication about workplace changes and expectations.

The considerable detail provided by respondents in their free text responses is indicative of their wish to have their voices heard. While free text responses are always limited by the inability to provide follow up questions (as would be the case in qualitative interviews), a strength of the free text responses in this study is the large and diverse sample size. There are limitations to the study findings as presented here. The survey question reported in this study specifically asked about challenges; hence, many of the comments are critical of systems and processes that caused health workers to experience difficulty and distress. Elsewhere in the survey, a minority of participants did identify helpful and positive aspects of their workplace environment. Further analyses of this data will help to identify the characteristics of safe and supportive workplaces and leaders. We also acknowledge that almost one third of survey participants did not answer this free text question.

## 5. Conclusions

Focusing on the workplace challenges experienced by healthcare workers is important, as there is consensus across much of the literature that pandemic preparedness in workplaces can alleviate mental health stress experienced by workers [39,45], as does having a supportive workplace [46]. Overall, participants in this study argued that health systems were unprepared for the pandemic and that there was limited support provided to frontline health workers. Better preparedness would include better communication between frontline health workers and management, especially if managers are removed from frontline work; adequate resource allocation (e.g., PPE supply); guidelines prepared ahead of time and reviewed regularly to ensure that updates match technological advances; and more attention to work-life balance. Better preparedness would lessen the detrimental effects of extreme work, by both reducing additional pandemic-specific work and reducing daily changes and communication of these changes. For many health workers, this did not occur as they struggled with the challenges of the COVID-19 pandemic. The overriding message conveyed by participants is the need for a safe workplace, where frontline workers feel valued and where there is a focus on retaining and supporting the workforce to ensure the provision of high-quality patient care.

## Figures and Tables

**Table 1:** Participants’ characteristics.

Characteristic	Frequency (*n* = 6679)	Percent (%)
Age (years)		
20–30	1449	21.7
31–40	1885	28.2
41–50	1522	22.7
>51	1823	27.2
**Gender**		
Male	1147	17.1
Female	5504	82.4
Other *	28	0.4
**Occupation**		
Nursing	2670	39.4
Medical **	1947	31.1
Allied Health ***	934	16.7
Administrative staff	407	6.2
Other roles ****	620	6.7

* responses of non-binary or prefer not to say. ** comprises Senior Doctor (*n* = 1032), Junior Doctor (*n* = 581), General Practitioner *n* = 334). *** comprises Dieticians (*n* = 64), Occupational Therapist (*n* = 160), Physiotherapist (*n* = 228), Psychologist (*n* = 124), Social Worker (*n* = 132), Paramedic (*n* = 82) Pharmacist (*n* = 144). **** comprises Allied Health unspecified and specified work groups with small numbers: dentistry, optometry, podiatry, medical imaging, scientists, technicians, support workers, students, leaders.

**Table 2:** From codes to key ideas to themes.

Code	Key Ideas Described	Theme
Work-life balance	Pervasiveness of COVID-19 in daily life	Increased workload and work-life imbalance
Workplace change	Trying to balance work and home roles and responsibilities Stretched staff resources and increased workload	
Staffing	Tensions of working from home and using telehealth Need for more flexibility	
Patient care	Disagreement about changes to the provision of patient care Concerns about compromised patient care Negotiating new restrictions on visitors	Following orders or caring for patients
Leadership	Lack of understanding by management	
Management	Not being listened to by leaders or managers	
Value	Feeling devalued by management	
Communication	Insufficient communication about guidelines Communication overload	Unpredictability, disruption, and inconsistency at work
Job Uncertainty	Confusion about conflicting or “mixed” messagingUnpredicability of role and changed practices	
Risk	Increased exposure to risk Insufficient protection	The right to be safe at work
Safety	Safety of workers not prioritised or valued	
PPE	Delays in access to PPE and PPE rationing	
Stress and anxiety	Feeling unsafe at work	
Preparedness	Reactive versus proactive Need for pre-planning	(Un)preparedness in the workplace
Information flow	Delayed or limited information provided to prepare	
Resources	Insufficient resources, planning, and training for pandemic response	
Health system	Lack of preparation across hospital, community, and primary care Unmet mental health needs	

## Data Availability

Data available upon reasonable request to the corresponding author.
